# Effect of planting methods and tillage practices on soil health and maize productivity

**DOI:** 10.3389/fpls.2024.1436011

**Published:** 2024-11-07

**Authors:** Peng Ju Gao, Hasnain Abbas, Fa Qiao Li, Guo Rong Tang, Ju Zhi Lv, Xun Bo Zhou

**Affiliations:** ^1^ Guangxi Key Laboratory of Agro-environment and Agro-products Safety, Key Laboratory of Crop Cultivation and Physiology, College of Agriculture, Guangxi University, Nanning, China; ^2^ Maize Research Institute of Guangxi Academy of Agricultural Sciences, Nanning, China

**Keywords:** straw treatment, sub-soiling, soil organic carbon, soil enzyme, yield

## Abstract

**Introduction:**

To increase the crop yield, the amount of agrochemicals used in field has increased in recent years. Moreover, indiscriminate use of chemical fertilizers has led to soil deterioration and compaction. Inclusion of straw and tillage practices to the field could play an important role in improving the soil quality and crop yield. Therefore, we hypothesized that combination of straw return and different tillage practices would result in improvement in soil health and crop productivity.

**Methods:**

Therefore an experiment was conducted a split plot design during 2018-2022. They were comprised of traditional planting with no straw return and straw return, accompanied by four different tillage methods: control (no tillage), rotary tillage (25 cm tillage depth), subsoiling (35 cm tillage depth), and subsoiling plus rotary tillage (35 + 25 cm tillage depth).

**Results:**

Results showed that subsoiling along with rotary tillage enhanced soil total nitrogen (TN) by 9.0%, soil organic carbon (SOC) 7.5%, soil microbial biomass carbon (MBC) 6.8%, soil catalase (S-CAT) 9.6%, soil urease (S-UE) 4.1%, soil cellulase (S-CL) 14.5%, soil sucrase (S-SC) 10.8% and maize yield 3.0% compared to no tillage.

**Discussion:**

Correlation analysis showed that (i) maize yield was significantly and positively correlated with S-SC, S-CL, S-UE, SOC, and TN. (ii) S-SC was significantly and positively correlated with TN, SOC, and MBC. (iii) TN was significantly and positively correlated with S-UE, and SOC was significantly and positively correlated with S-SC. It has been concluded that straw return coupled with subsoiling and rotary tillage is an appropriate approach to enrich soil nutrients, enzyme activities, and maize yield.

## 1 Introduction

Maize (*Zea mays* L.) is one of the most important food crops in the world (Nabeel et al., 2018); it is mainly grown as a double-season crop in southern parts of China and produces about 800 million tons of straw every year (Clare et al., 2015; Park et al., 2021). The main components of straw include cellulose, hemicellulose, lignin, and protein, which are important sources of soil organic matter. Straw burning is one of the traditional methods of straw disposal in agricultural production, and about 11% of straw is burned annually in southern China (Yu et al., 2017). Moreover, straw burning also seriously affects air quality (Li et al., 2020). Straw return to the soil could be a great strategy to reduce straw burning and reduce the emission of harmful gases such as CO_2_ and NO_2_ (Nan et al., 2020).

Plenty of chemical fertilizers have been used in arable lands for decades to fulfill global food demands. The long-term application of these chemical fertilizers could lead to soil acidification, reducing crop yields (Horrigan et al., 2002). Zheng et al. (2021) concluded that straw return to the field improved soil nutrients and enzyme activities, eventually improving soil structure and humus content. Straw return enhances the organic carbon content of topsoil (Chatterjee et al., 2018). Straw return may replace the majority of phosphorus and potassium fertilizers reduced the required amount of nitrogen fertilizers applied to arable land, eventually reducing soil erosion caused by improper utilization of chemical fertilizers (Li et al., 2024; Yin et al., 2018). Thus, several sustainable development techniques must be developed to handle excessive straw properly. Adding straw back into by the soil greatly increases the soil’s nutrients and crop yields (Liu et al., 2022; Gao et al., 2023). However, the conventional method of reusing straw in the field has several drawbacks, including the slow breakdown of the straw that prevents the nutrients from being properly utilized and the significant rise in pests and diseases.

Previous research has indicated that no-tillage can raise surface soil’s organic carbon content (Rahmati et al., 2020; Singh et al., 2020). With the continuous increase of global cultivated land area, the influence of no-tillage on soil physical environment is worth discussing. Many people have discussed the effect of no-tillage on crop yield (Pittelkow et al., 2015), C sequestration (Palm et al., 2014), and environmental quality (Mitchell et al., 2016), No-tillage management perturbs the soil less, leaves more residue on the soil surface than rotary tillage or even subsoiling, and affects soil properties differently than other farming systems. No-till management perturbs the soil less, leaves more residue on the soil surface than rotary tillage or even subsoiling, and affects soil properties differently than other farming systems. However, Cai et al. (2014) found that deep tillage can effectively protect soil structure, reduce soil bulk density, maintain soil water content, and provide a suitable soil environment for an increase in crop yield. Deep tillage also improves the soil organic carbon content of the 0-50 cm soil layer (Xu et al., 2019; Zhang et al., 2018). Nonetheless, persistent deep-tillage and no-tillage methods may worsen the degradation of soil physicochemical characteristics. Long-term no-tillage increases soil bulk density, which hinders plant root development and lowers agricultural yields (Kan et al., 2020). Reasonable tillage methods can improve soil quality, increase soil carbon and nitrogen storage, and promote sustainable utilization of cropland to mitigate the negative effects of deep tillage (He et al., 2019; Zhang et al., 2019). Conversely, showed that replacing no-tillage, rotary, and harrow tillage with deep tillage can boost soil carbon sequestration and crop yields (Tian et al., 2016).

Soil enzymes are crucial for the catalytic breakdown of organic matter, and their activity is an important indicator for assessing soil quality (Burns et al., 2013). There is a significant relationship between soil nutrients and enzyme activity, and soil enzymes play an active role in carbon and nitrogen breakdown (Zhao et al., 2016). The return of more straw to the field resulted in higher soil enzyme activity (Zhang et al., 2016). The straw altered the microbial community composition and soil structure, increasing the amount of organic matter and soil enzyme activity (Zhao et al., 2016). Zhao et al. (2019) found that straw return substantially improved urease activity in the 0-15 cm soil layer.

Only a few studies have been conducted on the combination of straw return and tillages practices effect on soil health and maize productivity. Therefore, we conducted this field experiment. The objective of this study was to evaluate the effects of different straw treatments and tillage practices on soil health and crop yield. The study result will provide a suitable agricultural practice for maize cultivation.

## 2 Materials and methods

### 2.1 Experimental site

**Figure 1:**
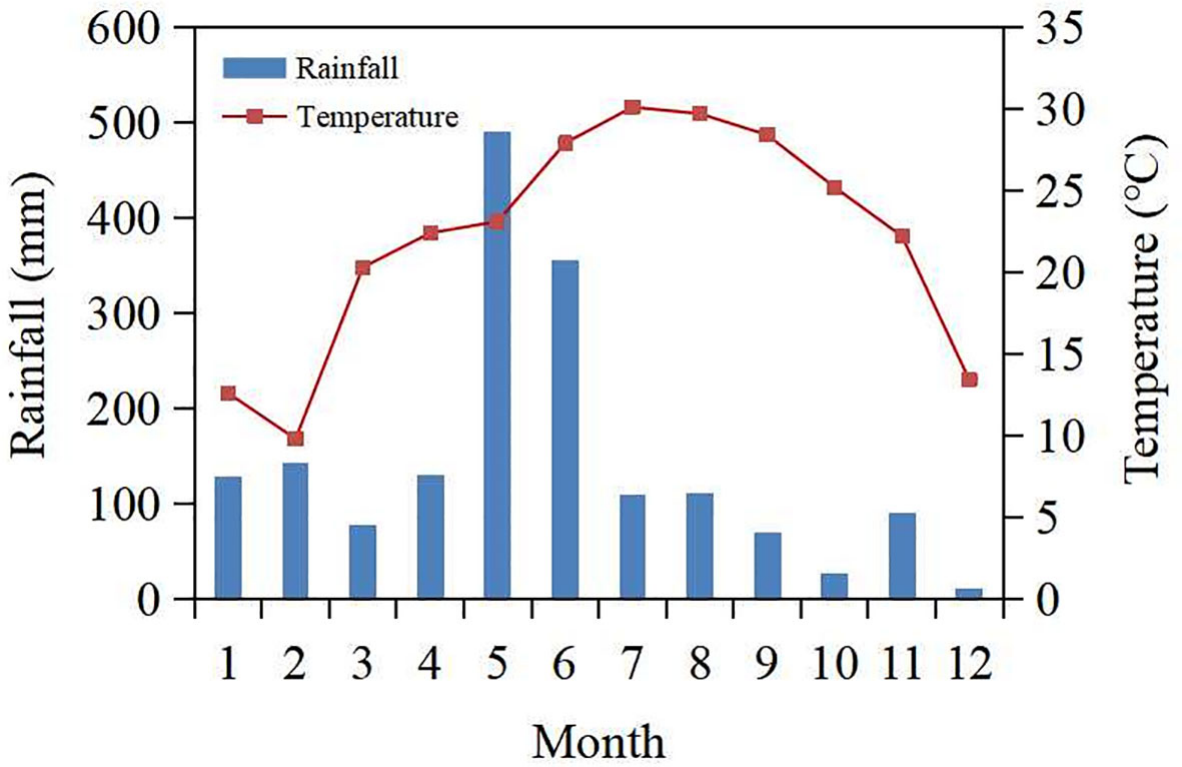
Monthly cumulative precipitation and temperature in 2022.

The current experiment was carried out at the Maize Research Institute of Guangxi Academy of Agricultural Sciences in Nanning, Guangxi, China (22°36′34′′N, 108°14′33′′E). The region has a humid subtropical monsoon climate, with average annual temperatures of 22.1°C and rainfall of 145.2 mm (**Figure 1**). The soil at the experiment site is loamy clay, with total nitrogen 0.9 g/kg, total phosphorus 1.5 g/kg, total potassium 33.3 g/kg, available phosphorus 65.3 mg/kg, available potassium 158.5 mg/kg, alkali-hydrolysable nitrogen 45.4 mg/kg, organic carbon 12.5 g/kg, pH 6.5, and bulk density 1.4 g/cm3 at the top 20 cm soil layer.

### 2.2 Experimental design

The results of this experiment were obtained from the 6^th^ year after 5 consecutive years of straw return to the field. The experiment adopted a split plot design with 8 treatments and 3 repetitions, the plot area was 20.8×11.2 m^2^ (233 m^2^). Straw return and traditional planting were placed in the main plot while no-tillage/control (NT), rotary tillage (RT), subsoiling (SS), and subsoiling plus rotary tillage (SS+RT) were placed in sub-plots. The depth of different tillage practices was as follows: control/no tillage (0 cm), rotary tillage (25 cm), sub-soiling (35 cm), subsoiling plus rotary tillage (35 + 25 cm). The previous crop in this experimental plot was also maize. The current maize crop was cultivated on ridges/beds with a row-row distance of 65 cm, while the plant-plant distance was 28 cm. Guidan-162 maize cultivar was used in this double-season experiment. The spring plantation was conducted on 20th March 2022, while the autumn was on 25th August 2022. The plant population was maintained at 52,500 plants/ha at the 3-4 leaf stage while the remaining plants were thinned out. Entek potassium sulfate, a slow-release fertilizer, was applied at the application 900 kg/ha before sowing (the recommended is 750-1000 kg/ha). It contains nitrogen, phosphorus, and potassium at a ratio of 21%, 7%, and 11%. Protective rows around the experimental field and large-scale production level were used to manage weeds, pests, and disease prevention and control.

### 2.3 Soil sampling

Five-point soil sampling method was used at the maturity (R6) stage to collect soil samples at four different soil depths, i.e., 0-10 cm, 10-20 cm, 20-30 cm and 30-40 cm. Thoroughly each depth soil sample was mixed respectively and passed through a 2 mm sieve, then every depth sample was further divided into two parts., One of which was immediately stored at -80°C in the laboratory to determined soil enzyme activities; the other portion was naturally dried, powders and sieved through 0.15 mm to determined soil nutrients.

### 2.4 Determination of soil nutrients

Soil total nitrogen (TN) was determined by the semi-micro Kjeldahl method (Li et al., 2008). Weigh 1.0000 g of air-dried soil over 0.150 mm in a boiling tube, add 2 g accelerant, 1 mL water, 5 mL concentrated H_2_SO_4_, cover a small curved neck funnel and let it stand for 24 h, then boil at 300°C for 1 h, turn gray or green for another 1 h, cool and set aside. Then, automatic titration was carried out using a nitrogen analyzer and a blank experiment.

Soil organic carbon was determined using the potassium dichromate volumetric external heating method (Chen et al., 2021). A total of 0.3000 g of air-dried soil that had passed through a 0.150 mm sieve was weighed into a boiling tube. Then, 5 mL H_2_SO_4_ and 5 mL of 0.8 mol/L K_2_Cr_2_O_7_ solution were added, respectively. Two blank test tubes were set up, and the curved neck of the small funnel was covered for 24 h. The mixture was heated to a boil for 1 h at 180-190°C, followed by an additional boiling period of 5 min, after which it was allowed to cool. After cooling, the contents of the test tube were washed into a conical flask with water, bringing the total volume to 60-70 mL. Two to three drops of o-phenanthroline were added, and the solution was titrated with 0.2 mol/L Fe_2_SO_4_, shaking while adding the titrant until the reaction was completed. The endpoint was indicated by a color change from orange-yellow to green to brick red. The volume of Fe_2_SO_4_ used in the titration was recorded.

The soil microbial biomass carbon (MBC) was determined by chloroform fumigation-potassium sulfate extraction method (Vance et al., 1987). 15.00 g fresh soil was weighed and put into a petri dish. After 7 days of closed dark culture in a dry tank, soil samples were extracted in 50 mL 0.5 mol/L K_2_SO_4_ for 30 min on ZWYR-4912 shaker (Shanghai Zhicheng Analytical Instrument Manufacturing Co., Ltd., Shanghai, China). Simultaneously with the extraction, another sub-sample (15.00 g fresh soil) were fumigated with chloroform for 24 h, All the fumigated soil samples were transferred to 150 mL triangular flasks. 50 mL of 0.5 mol/L K_2_SO_4_ (soil-water ratio of 1:4) was added, shaken for 30 min, and then filtered; meanwhile, a blank tube was prepared, finally titrated of all samples.

### 2.5 Determination of soil enzyme activity

Soil cellulase (S-CL), soil urease (S-UE), soil sucrase (S-SC), and soil catalase (S-CAT) were determined using Solarbio kits BC0155, BC0125, BC0145, BC0245, BC0105, and BC0165 (Science & Technology Co., Ltd., Beijing, China), as per the procedure of the manufacturer. S-CL: The production of 1 mg glucose per g soil sample per day was defined as an enzyme activity unit (U·g^-1^). S-UE: One unit of enzyme activity (U·g^-1^) was defined as 1 μg NH_3_-N produced per g soil sample daily. S-SC: 1 mg of reducing sugar per gram of soil per day at 37°C is an enzyme activity unit (U·g^-1^). S-CAT: 1 μg H_2_O_2_ degradation per g soil sample per day was defined as an enzyme activity unit (U·g^-1^).

### 2.6 Yield

At the R6 period, each plot was randomly selected as an area of 20 m^2^ to measure yield, which was converted to a kernel water content of 14%. 20 maize plants were chosen from each area for indoor testing, and the number of rows of ears, the number of grains in the rows, and the weight of 1,000 grains were counted.

### 2.7 Statistical analysis

All data were subjected to two-way ANOVA using SPSS 21.0 software (IBM Inc., Chicago, IL, USA), the significantly differences was determined by the least significant difference (LSD) at *P* = 0.05. All graphs were processed using OriginPro 2021 (Origin Lab Corporation, Northampton, MA, USA).

## 3 Results

### 3.1 Soil total nitrogen

**Figure 2:**
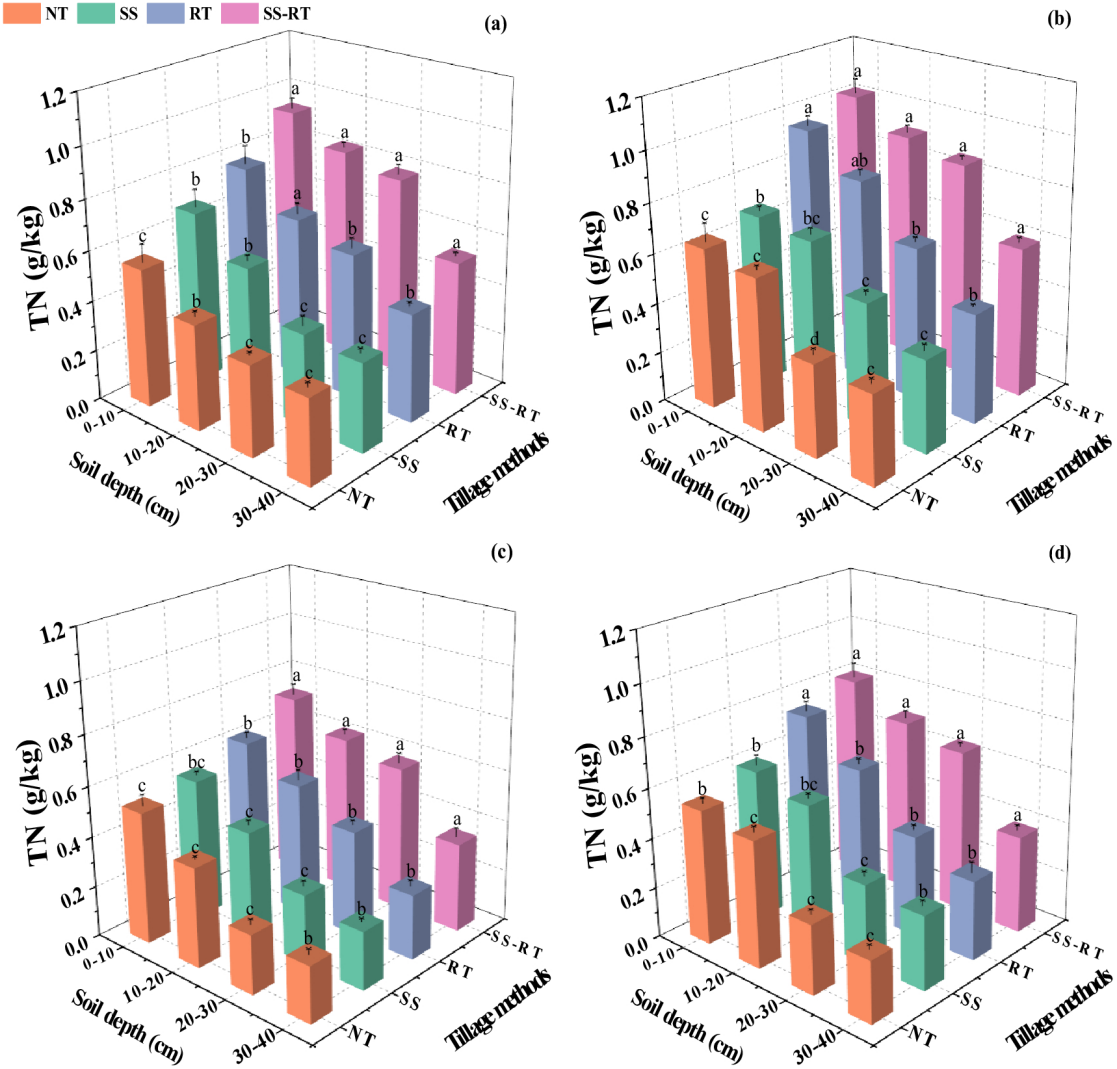
Effect of straw return combined with tillage methods on soil total nitrogen under dual-cropping system during spring seasons with traditional planting **(A)** and straw return **(B)**, and in autumn seasons with traditional planting **(C)** and straw return **(D)**. Results represent the mean ± SD (n = 3). Different letters indicate significant differences between soil layers (*P* < 0.05). SS-RT, subsoiling and rotary tillage; RT, rotary tillage; SS, subsoiling; NT, no-tillage.

Analysis of the data from both seasons under the straw and tillage treatments indicated that TN content was significantly affected in both spring and fall and was higher in the spring than in the fall (**Figure 2**). In the spring, straw return significantly increased TN content by 11.5% compared to that under traditional planting. The TN content increased by 9.0%, 13.8%, 12.1%, and 13.18% in straw return compared to SS-RT, RT, SS, and NT treatments under traditional planting, respectively. Under both traditional planting and straw return, both showed the highest TN content in the 0-10 cm soil layer in the SS-RT treatment, which was 1.0 and 0.9 g/kg, respectively, and was significantly higher in SS-RT than RT, SS, and NT among all treatments. TN content was 0.5 and 0.4 g/kg under straw return and traditional planting, respectively, during the fall fertility period. TN content decreased with the increase of soil depth. The SS-RT treatments had the highest TN content under straw return, which was 8.52% higher than RT, SS, and NT treatments under 0-10 cm and 10-20 cm soil layers, respectively, 25.7%, 33.5% and 15.2%, 22.3% and 27.8%. Under straw return and traditional planting, all soil layers showed that straw return was higher than traditional planting; in the 0-10 cm soil layer, TN content of SS-RT, RT, SS, and NT treatments under straw return was increased by 10.6%, 16.7%, 8.0% and 3.2%, respectively, compared to traditional planting, and in 10-20 cm soil layer, it was increased by 11.2%, 12.4%, respectively, 21.2% and 22.0%.

### 3.2 Soil organic carbon

**Figure 3:**
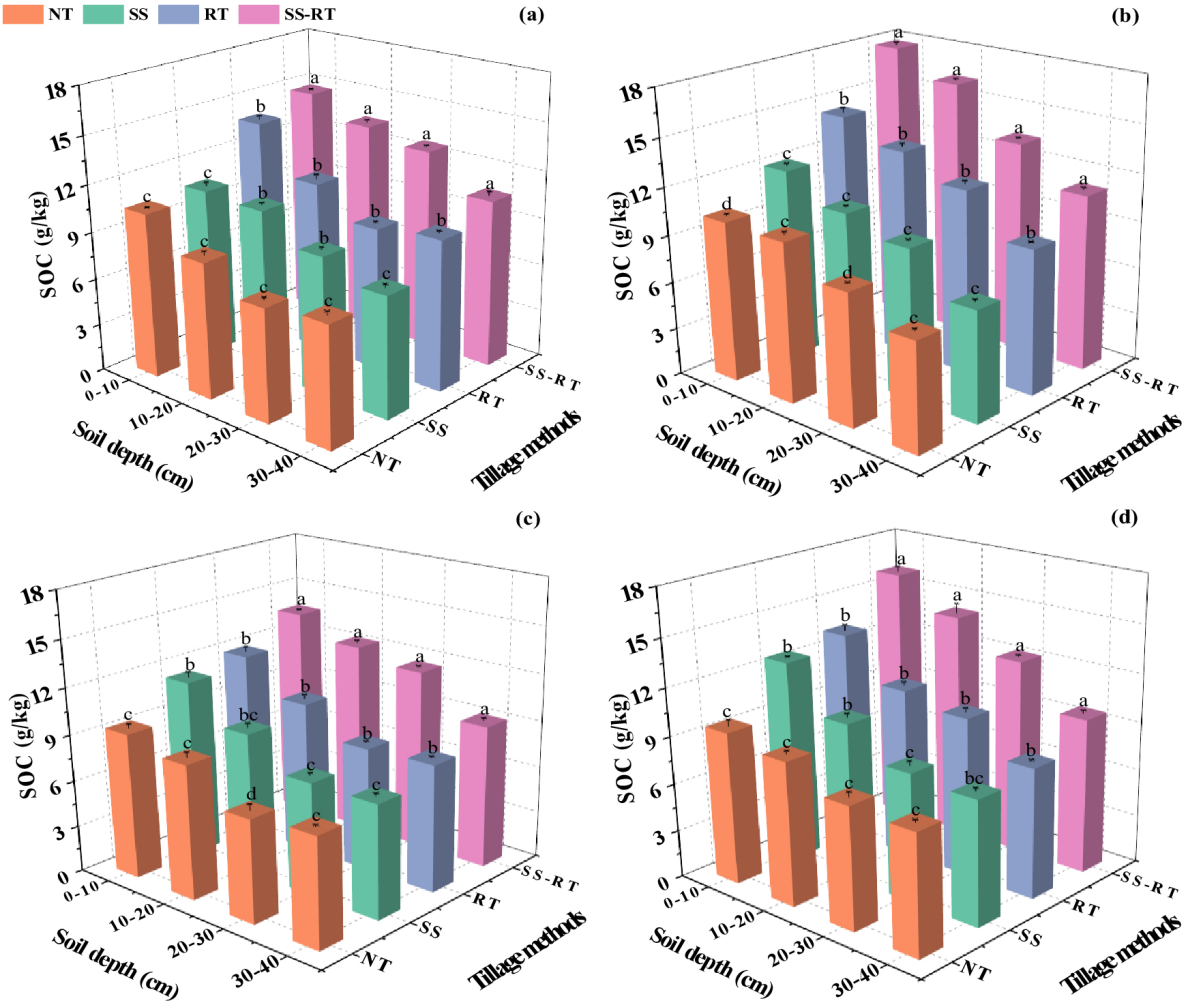
Effect of straw return combined with tillage methods on soil organic carbon under dual-cropping system during spring seasons with traditional planting **(A)** and straw return **(B)**, and in autumn seasons with traditional planting **(C)** and straw return **(D)**. Results represent the mean ± SD (n = 3). Different letters indicate significant differences between soil layers (*P* < 0.05). SS-RT, subsoiling, and rotary tillage; RT, rotary tillage; SS, subsoiling; NT, no-tillage.

Soil organic carbon (SOC) content decreased gradually with increasing soil depth, and straw application increased SOC content (**Figure 3**). In spring, the SOC content increased by 8.4% under straw return compared to traditional planting. Under traditional planting, no significant change in SOC content in RT, SS, and NT treatments existed in the four soil horizons. However, the overall performance of SS-RT treatments showed significantly higher SOC content than that of RT, SS, and NT. The SOC content of SS-RT treatment was highest at 0-10 cm, 10-20 cm, 20-30 cm, and 30-40 cm under straw return, which was 42.9%, 37.8%, 37.4%, and 38.1% higher than that of NT treatment, respectively. The overall SOC content of the treatments in fall was slightly lower than that in spring, and the SOC content of the RT and SS treatments in the 0-10 cm, 10-20 cm, 20-30 cm, and 30-40 cm soil horizons under traditional planting and straw return did not change significantly. All the SS-RT treatments had the highest SOC under straw return, significantly increasing by 19.4%, 26.4%, and 35.3% over RT, SS, and NT treatments, respectively. SOC was significantly increased by 10.3%, 9.9%, 8.4%, and 6.1% in SS-RT, RT, SS, and NT treatments under straw return compared to traditional planting.

### 3.3 Soil microbial biomass carbon

**Figure 4:**
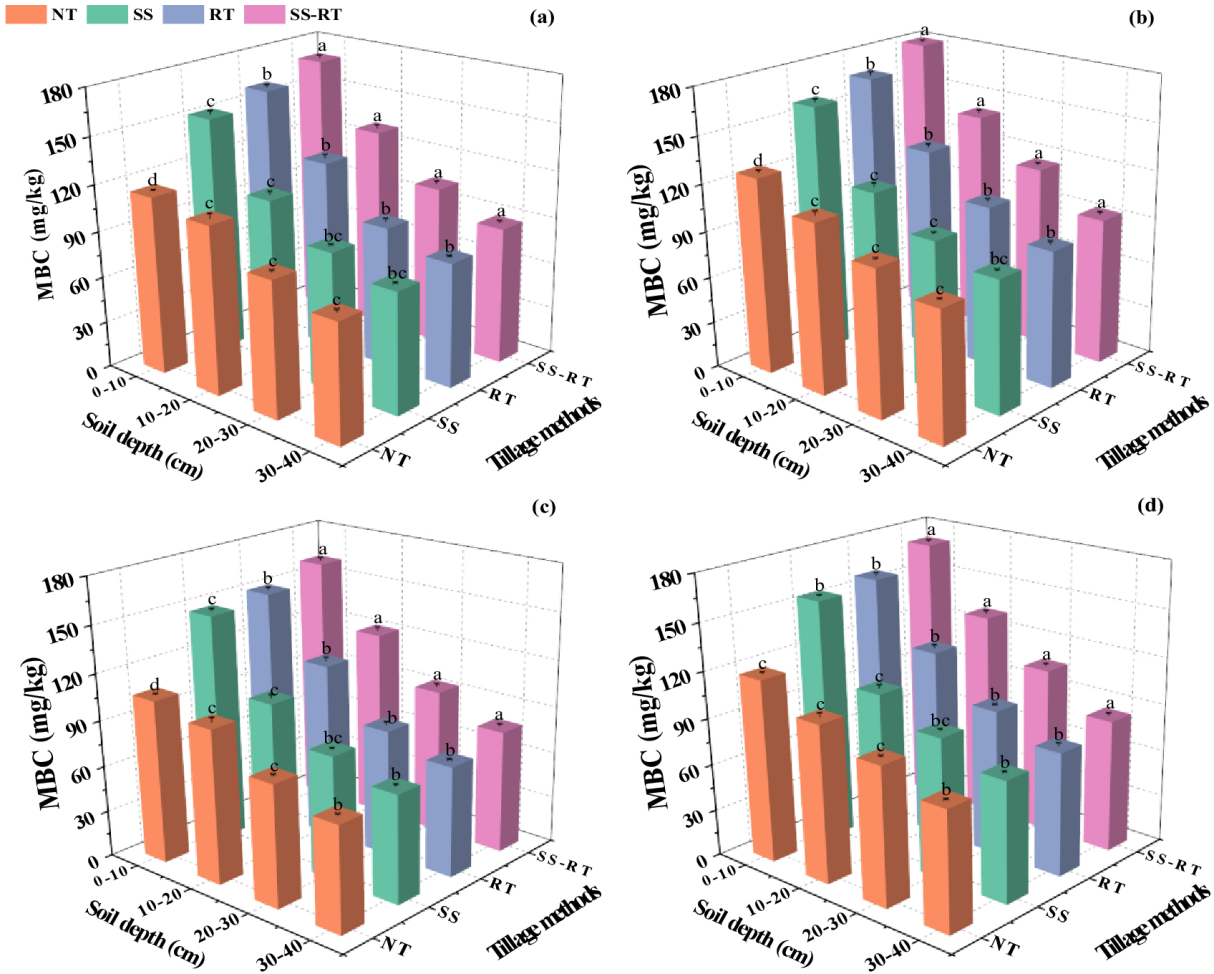
Effect of straw return combined with tillage methods on soil microbial biomass carbon under dual-cropping system during spring seasons with traditional planting **(A)** and straw return **(B)**, and in autumn seasons with traditional planting **(C)** and straw return **(D)**. Results represent the mean ± SD (n = 3). Different letters indicate significant differences between soil layers (*P* < 0.05). SS-RT, subsoiling, and rotary tillage; RT, rotary tillage; SS, subsoiling; NT, no-tillage.

The MBC content under straw return was 8.0% higher than that under traditional planting (**Figure 4**). The differences in MBC content between SS and NT treatments in all four soil depths were insignificant under straw return and conventional planting. MBC content was increased by 5.7%, 4.40%, 4.5%, and 9.1% in SS-RT, RT, SS, and NT treatments, respectively, and by 6.9%, 5.4%, 4.0%, and 1.5% in 10-20 cm soil layer, respectively, compared to traditional planting under straw return. The trend of MBC content across treatments in the fall was consistent with that in the spring. MBC content was significantly increased by 7.8%, 13.7%, and 21.6% in SS-RT treatment under straw return compared to RT, SS, and NT treatments. Straw return was 7.1% (SS-RT), 7.2% (RT), 5.8% (SS), and 6.7% (NT) higher than traditional planting, respectively.

### 3.4 Soil enzyme activity

**Figure 5:**
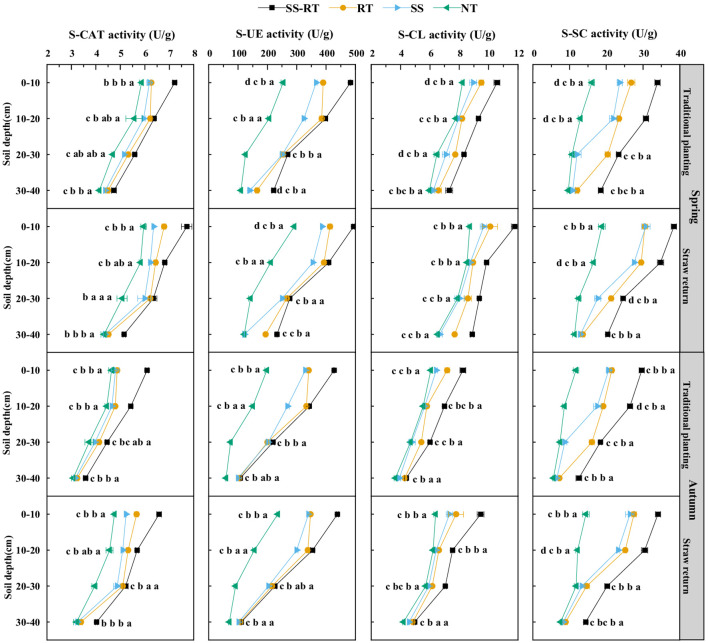
Effect of straw return and tillage methods on soil enzyme activity. Results represent the mean ± SD (n = 3). Different letters in the row indicate significant differences in tillage practices, *P* < 0.05, SS-RT, subsoiling, and rotary tillage; RT, rotary tillage; SS, subsoiling; NT, no-tillage.

In **Figure 5**, the combined application of straw return and tillage methods improved soil enzyme activities for both seasons. Averaged across four soil depths, the straw return increased S-CAT, S-UE, S-CL, and S-SC activities by 6.8%, 4.8%, 9.9%, and 15.0% in spring and 8.4%, 5.1%, 12.0%, and 18.8% in autumn. At the same time, the soil enzyme activity trend was SS-RT > RT > SS > NT under SS-RT, RT, SS, and NT in both seasons, respectively. The S-CAT, S-UE, S-CL, and S-SC activities were higher in the SS-RT treatments than in RT, SS, and NT in the 0-10 cm and 10-20 cm soil layers under straw return, but the differences were not significant among the RT and SS treatments. Compared to NT, SS-RT, RT, and SS increased S-CAT activity by 17.5%, 9.7%, and 6.1%, S-UE activity by 46.1%, 39.2% and 32.8%, S-CL activity by 20.0%, 9.5% and 5.9%, and S-SC activity by 53.0%, 42.5% and 34.5%, respectively. Moreover, S-CAT, S-UE, S-CL, and S-SC activities were reduced by 20.3%, 20.3%, 28.5%, and 20.6%, respectively, in fall compared with spring.

### 3.5 Yield and yield components

The two-season maize trial showed that the number of grains in the ear, the kernel weight in thousand kernels, and the yield were closely related to straw treatment and tillage methods (**Table 1**). The number of grains in ears, the kernel weight in thousand kernels, and the yield were significantly higher in spring and fall under straw-returned conditions. Compared to traditional planting, under straw return, the number of grains in the ear, thousand-grain weight, and yield increased by 4.9%, 4.1%, and 3.4%, respectively. It improved by 2.7%, 7.3%, and 3.2% in the fall. The maize thousand-grain weight and yield of SS-RT treatment were higher than those of the other three treatments, respectively. For instance, the SS-RT treatment in the spring increased the thousand-grain weight by 1.8%, 6.9%, and 12.9% compared to RT, SS, and NT, respectively; the yield was increased by 3.3%, 10.4%, and 15.3% over RT, SS, and NT, respectively. The average results of spring and fall seasons showed that the number of spikes, thousand-grain weight, and yield of straw return were 3.8%, 5.7%, and 3.3% higher than traditional planting, respectively. Yields of SS-RT, RT, and SS treatments were 14.8%, 12.0%, and 4.6% higher than NT, respectively. Spring maize yield was higher than fall maize at 8599 kg/ha and 7906 kg/ha, respectively.

**Table 1:** Effects of straw return and tillage methods on yield components of maize.

			KNE (grain/ear)			TGW (g)			Yield (kg/ha)		
			Spring	Autumn	Mean	Spring	Autumn	Mean	Spring	Autumn	Mean
Traditional planting	NT		396^d^ ± 2.65	446^d^ ± 3.08	421^d^ ± 2.86	280.8^d^ ± 5.25	263.2^c^ ± 3.75	272.0^d^ ± 3.59	7769^d^ ± 39.14	7117^d^ ± 40.61	7443^d^ ± 49.88
	SS		434^c^ ± 3.61	455^c^ ± 3.30	444^c^ ± 2.37	310.7^c^ ± 3.73	267.2^c^ ± 2.27	289.0^c^ ± 2.98	8245^c^ ± 45.32	7461^c^ ± 29.02	7852^c^ ± 20.59
	RT		468^b^ ± 4.00	477^b^ ± 6.38	473^b^ ± 2.38	326.8^b^ ± 1.44	290.0^b^ ± 2.25	308.4^b^ ± 1.67	8735^b^ ± 56.52	8081^b^ ± 48.17	8408^b^ ± 50.64
	SS-RT		504^a^ ± 8.72	501^a^ ± 5.63	503^a^ ± 2.79	336.6^a^ ± 1.93	300.0^a^ ± 5.76	318.1^a^ ± 3.69	9061^a^ ± 52.77	8459^a^ ± 38.33	8760^a^ ± 18.87
Straw return	NT		416^d^ ± 4.00	459^d^ ± 2.37	438^d^ ± 0.83	309.3^c^ ± 4.20	296.0^c^ ± 1.99	302.6^c^ ± 1.86	7932^d^ ± 53.33	7479^d^ ± 17.18	7705^d^ ± 18.19
	SS		476^c^ ± 2.65	469^c^ ± 2.50	473^c^ ± 1.30	320.1^b^ ± 4.39	298.2^bc^ ± 3.99	309.2^b^ ± 0.25	8364^c^ ± 36.43	7698^c^ ± 26.32	8031^c^ ± 30.64
	RT		488^b^ ± 2.65	486^b^ ± 3.03	487^b^ ± 0.75	338.6^a^ ± 3.24	304.1^ab^ ± 3.73	321.4^a^ ± 3.20	9205^b^ ± 43.21	8406^b^ ± 16.40	8806^b^ ± 29.06
	SS-RT		514^a^ ± 4.00	517^a^ ± 1.96	516^a^ ± 2.62	340.8^a^ ± 2.60	310.0^a^ ± 3.63	325.4^a^ ± 2.41	9484^a^ ± 48.51	8548^a^ ± 39.84	9016^a^ ± 35.98
*P* value											
ST		0.0040		0.0255	0.0103	0.0264	0.0007	0.0053	0.0050	0.0005	0.0010
TM		0.0001		0.0001	0.0001	0.0001	0.0001	0.0001	0.0001	0.0001	0.0001
ST×TM		0.0006		0.1281	0.0003	0.0001	0.0022	0.0001	0.0001	0.0001	0.0001

KNE, TGW, and Yield represent the number of grains in the ear, thousand kernel weight, and yield, respectively. Results represent the mean ± SDs (n = 3). Different letters in the same column indicate significant differences, *P* < 0.05.

### 3.6 Correlation analysis

**Figure 6:**
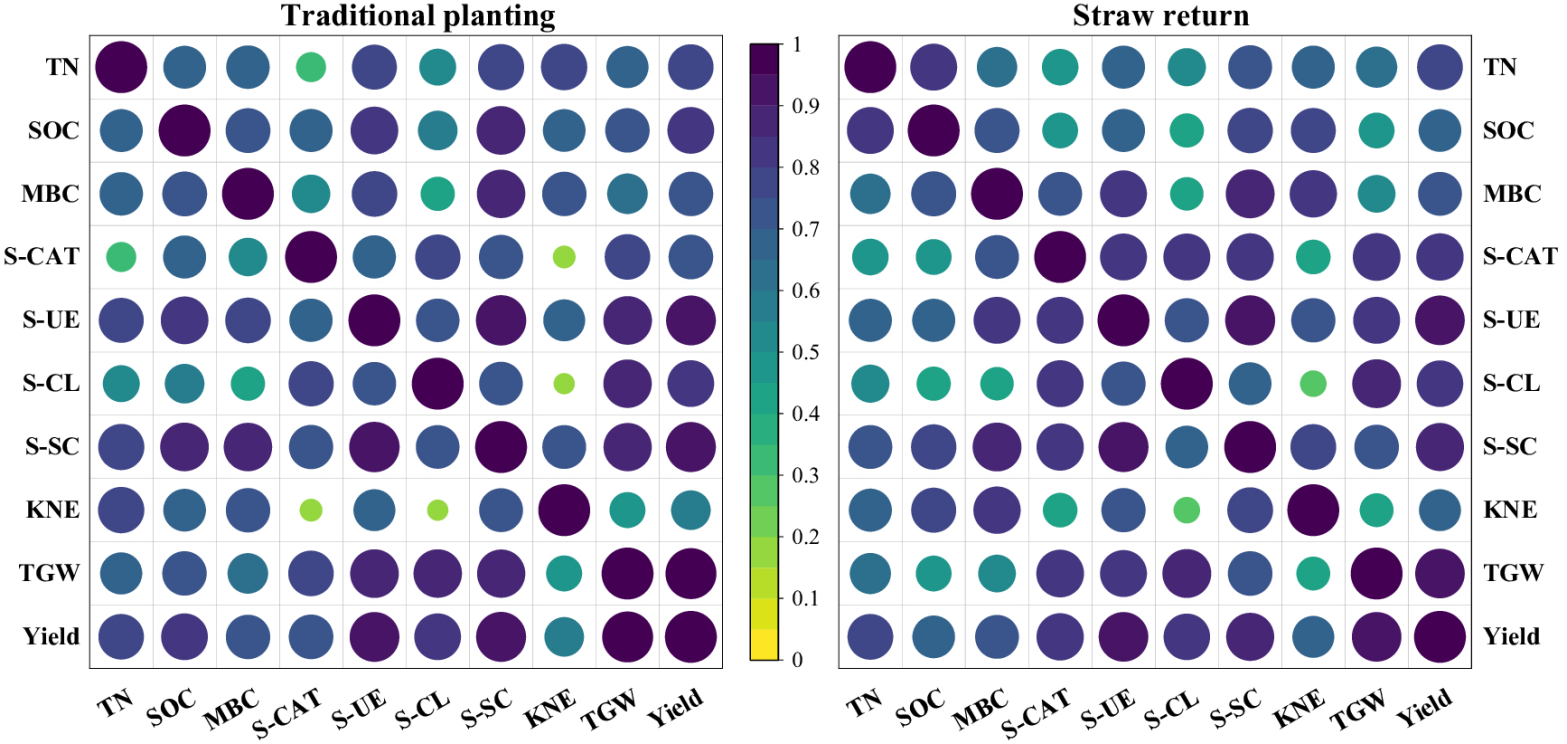
Correlation analysis among soil nutrients, enzyme activities, yield, and yield components under straw return combined with tillage methods. TN, SOC, MBC, S-CAT, S-UE, S-CL, S-SC, KNE, TGW, and Yield stand for total soil nitrogen, soil organic carbon, soil microbial carbon, soil catalase, soil urease, soil cellulase, soil sucrase, grain number per ear, thousand-grain weight and yield, respectively.

The results of correlation analysis showed that yield was significantly and positively correlated with TWG, S-SC, S-CL, S-UE, SOC, and TN (*P* < 0.01), the correlation coefficients of TWG and KNE showed weaker association with Pn for both straw return and traditional planting (**Figure 6**). Likewise, the TWG was positively correlated with S-SC,S-CL,S-UE,SOC (*P* < 0.01). However, the correlation coefficients of TWG and KNE showed a weaker association. Under straw return and traditional planting, S-SC was significantly and positively correlated with TN, SOC, and MBC (*P* < 0.01). Moreover, the correlation coefficients of S-CAT and TN showed weaker association under the traditional planting; apart from this, TN was significantly and positively correlated with S-UE (*P* < 0.01), SOC was significantly and positively correlated with S-SC (*P* < 0.01), the correlation coefficients of MBC and S-CL showed a weaker association. The analysis showed that the Soil nutrients, enzyme activities, and cultivation systems showed variable relationships.

## 4 Discussion

Straw return can increase total nitrogen, organic carbon and microbial carbon in the soil, which has far-reaching impacts on the sustainable development of agriculture (Khan et al., 2019). In the long term, it seems that straw return can store water and conserve moisture, promote the growth of the maize root system, and provide a favorable environment for crop development (Ma et al., 2020). Under the action of microorganisms, straw becomes humus, increasing organic and microbial carbon content and improving soil nutrient content (Yang et al., 2022). Therefore, straw return is a good way to utilize straw. Tillage method are a common agronomic measure, no-tillage has the potential to promote soil carbon sequestration and reduce greenhouse gas emissions (Sasode et al., 2020), some experts have also found that the dry matter accumulation and yield of crops treated with no-tillage are higher (Kamboj et al., 2017). However, not all situations are perfect, prolonged no-tillage and over-tillage can lead to soil compaction and nutrient loss. For instance, nitrogen in the soil can leach into groundwater in the form of NO_3_^-^ (Zhou et al., 2020). The results of this study showed that straw return increased SOC by 8.38% compared to conventional planting, the SOC sequestration was increased by straw return under 0-10 cm and 10-20 cm soil layers. The straw return can increase soil microbial species and numbers, favoring SOC decomposition and sequestration (Fang et al., 2018; Ma et al., 2020). The MBC participates in many biochemical reactions in the soil system and maintains the ecological balance of the soil as an integral part of it (Guo et al., 2016). This study found that straw return in conjunction with tillage methods can increase the soil microbiome carbon content, consistent with (Zhao et al., 2016). In general terms, the greater changes in SS-RT and RT than in SS and NT under straw return may be related to the acceleration of soil respiration and the degradation of small molecule organic matter by tilling and rotary tillage after straw return (Ning et al., 2009).

Soil enzymes are widely present in soil and are important organic soil components (Salam et al., 1998). Zhao et al. (2016) found that long-term straw return can increase soil enzyme activities, Zhang et al. (2016) showed that four consecutive years of straw return increased soil urease and invertase activities, which were closely related to SOC. Our study showed that catalase, urease, cellulase, and sucrase behaved similarly in the 0-40 cm soil layer, all of which showed a gradual decrease in enzyme activity as the depth of the soil layer increased. Their activities may be related to the metabolic activities of soil microorganisms after the straw return (Yang et al., 2016). The soil urease, cellulase, and sucrase activities were significantly higher than those of other treatments under 0-10 cm and 10-20 cm soil layers, which might be related to the depth and content of straw mixed into the soil (Li et al., 2017). Soil enzyme activities were also affected by tillage methods, with the highest soil carbon content in the SS-RT, RT, and SS treatments providing more energy to the microorganisms, resulting in increased soil urease, cellulase, and sucrase activities compared to NT. In addition, RT and SS treatments disturbed the soil structure less and improved the soil environment suitable for microbial growth. Bielińska and Mocek-Płóciniak (2012) state that suitable tillage stimulates enzyme activity, similar to the present study, and that enzyme activity is higher in shallow soils. We found that RT-SS, RT, and SS treatments had higher enzyme activities.

Tillage is an important farm management system that can be used to address soil erosion from waterlogging and stabilize crop yields (Campiglia et al., 2015; Sang et al., 2016); different planting methods significantly affected crop agronomic traits and yield. The data parameters recorded by manual transplanting were comparable to those recorded by mechanical transplanting, and both were higher than those recorded by no-tillage treatment (Meena et al., 2016). In this study, SS-RT, RT, and SS significantly increased maize yield compared to NT. This may be related to the tillage method that increased soil water content. In the tropics, grain yield is closely related to crop water supply, and maize growth relies heavily on soil water storage after rainfall due to higher temperatures in Guangxi, where soil moisture evaporation exceeds precipitation (Guan et al., 2015). Thus, improved soil water content by SS-RT, RT, and SS resulted in higher maize yields than NT. Our study showed that straw return combined with tillage method increased the stability of maize yields, probably because these methods increased SOC sequestration and played a role in yield stabilization and improvement. Accordingly, the results of our study emphasize the importance of tillage methods and straw return increasing soil carbon and maintaining high and stable yields (Liska et al., 2014). Straw return influences the nitrogen dynamics within the soil, which is crucial for crop productivity. Different straw return led to significant increases in soil nitrogen content (Yang et al., 2024). The increase in soil nitrogen directly correlates with maize yield, particularly in the tilling layers, where the contribution rates to yield were 31.6%-43.1%. The results indicated that straw return combine tillage treatments increased nitrogen content, contributing to increased maize yield from SS-RT to NT treatments. This highlights the role of nitrogen enrichment through straw return in enhancing maize productivity.

## 5 Conclusions

This study clarified the effects of straw return and tillage methods on soil nutrients, enzyme activities and maize yield, and provided reliable technical support for maize cultivation in Guangxi. The annual soil TN, SOC, MBC, S-CAT, S-UE, S-CL and S-SC contents of straw return were significantly increased by 13.5%, 3.1%, 7.1%, 7.5%, 5.2%, 12.1% and 5.2% compared with traditional planting, respectively. Straw return combined with SS-RT improved soil quality, increased soil nutrients and further increased yield. Under straw return, the annual maize yield under SS-RT treatment was the highest, which was 2.9% higher than that under traditional planting. Therefore, the combination of straw return and SS-RT is the best strategy. The fourth consecutive year of straw return improved soil quality in Guangxi. But reducing straw return amount and straw crushing degree in the future needs further research.

## Data Availability

The original contributions presented in the study are included in the article/supplementary material. Further inquiries can be directed to the corresponding author.
